# DNA Amplification and Detection Made Simple (Relatively)

**DOI:** 10.1371/journal.pbio.0040222

**Published:** 2006-06-13

**Authors:** Mary Hoff

Twenty-three years ago, a man musing about work while driving down a California highway revolutionized molecular biology when he envisioned a technique to make large numbers of copies of a piece of DNA rapidly and accurately. Known as the polymerase chain reaction, or PCR, Kary Mullis's technique involves separating the double strands of a DNA fragment into single-strand templates by heating it, attaching primers that initiate the copying process, using DNA polymerase to make a copy of each strand from free nucleotides floating around in the reaction mixture, detaching the primers, then repeating the cycle using the new and old strands as templates. Since its discovery in 1983, PCR has made possible a number of procedures we now take for granted, such as DNA fingerprinting of crime scenes, paternity testing, and DNA-based diagnosis of hereditary and infectious diseases.

As valuable as conventional PCR is, it has limits. Heat is required to separate the DNA and cooler temperatures are needed to bind the primer to the strands, so the reaction chamber must repeatedly cycle through hot and cold phases. As a result, the technique can only be performed in laboratories using sophisticated equipment.

Now Olaf Piepenburg, Niall Armes, and colleagues have come up with a new approach to DNA amplification that can be carried out at a constant temperature, using only a tiny amount of DNA, without elaborate equipment. Called recombinase polymerase amplification (RPA), the technique opens the door to dramatically extending the application of DNA amplification in fieldwork and in laboratories where PCR machines are not available.

RPA uses five main ingredients: a sample of the DNA to be amplified; a primer–recombinase complex, which initiates the copying process when it attaches to the template; nucleotides from which to form the new strands; a polymerase, which brings them together in the right order; and single-stranded DNA-binding proteins (SSBs), which help keep the original DNA from zipping back together while the new DNA is being made. The primer–recombinase complex is able to attach to the double-stranded DNA, eliminating the need to heat the mixture. After the complex is in place, it disassembles, allowing the DNA polymerase to begin synthesizing a new strand of DNA complementary to the template, while the SSBs attach to and stabilize the displaced strand. Under the right conditions—a precise milieu of process-regulating chemicals—the process automatically repeats, resulting in an exponential increase in the DNA sample.

The researchers tested the sensitivity, specificity, and speed of RPA by using it to amplify three kinds of human DNA, as well as DNA from *Bacillus subtilis*. They found it to be rapid and accurate. However, they also noted that using RPA to detect the presence of a specific type of DNA—for example, that of a specific pathogen—was complicated by the fact that, at low or zero concentration, the primer also produced an artifact effect (akin to some similar PCR artifacts). To counteract this, the researchers developed a probe-based detection method that causes the sample to glow in the presence of the DNA being tested for, but not in the presence of primer alone.

To demonstrate the usefulness of the new system, the researchers used it to test for the presence of methicillin-resistant *Staphylococcus aureus* (MRSA), a disease-causing bacterium known as a “superbug” because it is unharmed by penicillin antibiotics. They found that RPA could detect fewer than ten copies of MRSA DNA. It could also determine the presence of three different genotypes of MRSA, and distinguish them from a methicillin-sensitive S. aureus strain.

How easy would it be to apply such a test in real-life situations? The researchers demonstrated one possible approach by encapsulating the entire process in a dipstick that could be used in the field to detect the presence of a pathogen.

As great as the potential of RPA is for making DNA amplification and detection easier and more broadly applicable, that's not its only value. The researchers noted that the reaction environment they developed also provides a framework for improving understanding of recombination and the application of DNA hybridization.

## 

**Figure:**
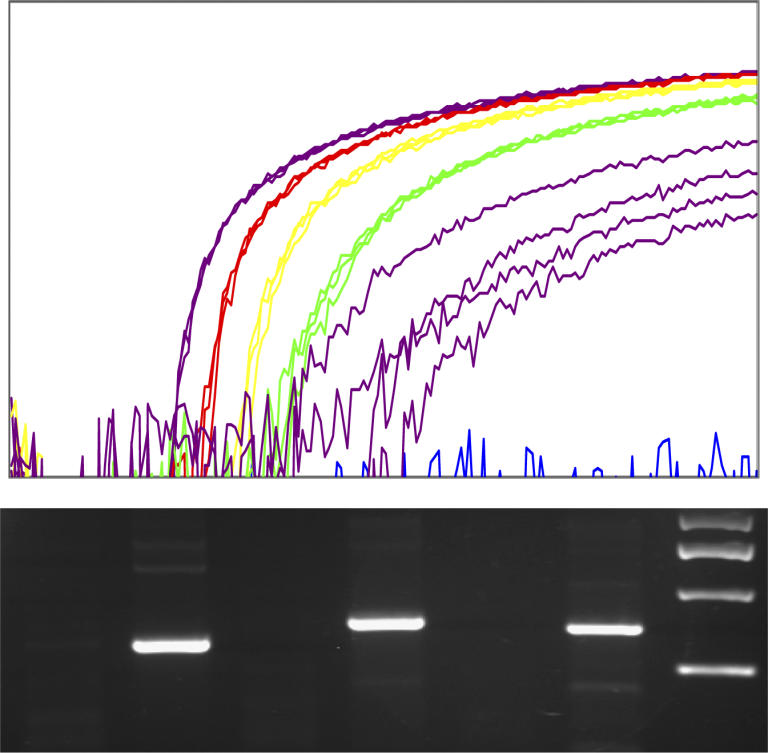
Reactions monitored in real-time with a fluorescent probe demonstrate a linear time-dependent response to copy number (top). A wide variety of targets in complex DNA can be amplified using specific primer pairs (bottom).

